# Performance and user evaluation of a novel capacitance-based automatic urinometer compared with a manual standard urinometer after elective cardiac surgery

**DOI:** 10.1186/s13054-015-0899-4

**Published:** 2015-04-21

**Authors:** Anton Eklund, Martin Slettengren, Jan van der Linden

**Affiliations:** Department of Cardiothoracic Surgery and Anesthesiology, Karolinska University Hospital, SE-17176 Stockholm, Sweden; Department of Molecular Medicine and Surgery, Karolinska Institutet, SE-17177 Stockholm, Sweden

## Abstract

**Introduction:**

In the intensive care setting, most physiologic parameters are monitored automatically. However, urine output (UO) is still monitored hourly by manually handled urinometers. In this study, we evaluated an automatic urinometer (AU) and compared it with a manual urinometer (MU).

**Methods:**

This prospective study was carried out in the intensive care unit of a cardiothoracic surgical clinic. In postoperative patients (n = 34) with indwelling urinary catheters and an expected stay of 24 hours or more, hourly UO samples were measured with an AU (Sippi, n = 220; Observe Medical, Gothenburg, Sweden) or an MU (UnoMeter™ 500, n = 188; Unomedical, Birkerød, Denmark) and thereafter validated by cylinder measurements. Malposition of the instrument at the time of reading excluded measurement. Data were analyzed with the Bland-Altman method. The performance of the MU was used as the minimum criterion of acceptance when the AU was evaluated. The loss of precision with the MU due to temporal deviation from fixed hourly measurements was recorded (n = 108). A questionnaire filled out by the ward staff (n = 28) was used to evaluate the ease of use of the AU compared with the MU.

**Results:**

Bland-Altman analysis showed a smaller mean bias for the AU (+1.9 ml) compared with the MU (+5.3 ml) (*P* <0.0001). There was no statistical difference in measurement precision between the two urinometers, as defined by their limits of agreement (±15.2 ml vs. ±16.6 ml, *P* = 0.11). The mean temporal variation with the MU was ±7.4 minutes (±12.4%), and the limits of agreement were ±23.9 minutes (±39.8%), compared with no temporal variation with the AU (*P* <0.0001). The ward staff considered the AU easy to learn to use and rated it higher than the MU (*P* <0.0001).

**Conclusions:**

The AU was not inferior to the MU and was significantly better in terms of bias, temporal deviation and staff opinion, although the clinical relevance of these findings may be open to discussion.

**Electronic supplementary material:**

The online version of this article (doi:10.1186/s13054-015-0899-4) contains supplementary material, which is available to authorized users.

## Introduction

Most vital parameters, including heart rate, blood pressure, temperature and arterial saturation, are continually monitored by automatic instruments in today’s surgical and intensive care units (ICUs). Moreover, the fluid input is usually also monitored with automatic volume pumps and syringe pumps connected to the patient monitoring system. On the contrary, urine output (UO), the predominant factor of the total fluid output, is usually monitored by manual registration [1]. Implementing an automatic urinometer (AU) instead of a manual urinometer (MU) may as such reduce human errors and ease the workload of the ward staff. It may also lead to UO measurement being used more frequently on less well-staffed wards, where the staff does not have the time to manually register the patients’ diuresis hourly. An AU could also give physicians a better means to monitor and plan patient fluid therapy and as such be beneficial for the patients, although this has yet to be proven. Measuring hourly diuresis is important, such as during and after surgery, because it may help to detect early signs of renal dysfunction, and specifically acute kidney injury, a common complication after major surgery and during hemodynamic instability [2,3].

The aim of this study was to evaluate the performance of a new AU that continuously monitors UO via capacitance measurements and compare it with an MU with regard to bias, precision, temporal deviation and standard evaluation of diagnostics and staff opinion.

## Material and methods

Adult patients scheduled for cardiac surgery in the cardiothoracic department of Karolinska University Hospital, Stockholm, Sweden, were enrolled in the study after they provided their written consent to participate. Ethical approval was obtained from the regional ethical review board in Stockholm (decision reference number 2012/31-31/2), and the study was carried out in compliance with the Declaration of Helsinki [4]. No exclusion criteria were used. Patients received an indwelling urinary catheter in the operating room after induction of anesthesia. After arrival to the ICU, patients were connected to either the new AU (Sippi; Observe Medical, Gothenburg, Sweden) (Additional files 1 and 2) or a standard MU (UnoMeter™ 500; Unomedical, Birkerød, Denmark). The urinometers were evaluated during two separate time periods, with the MU being evaluated first to reflect the routine UO measurement performance, followed by the AU evaluation. No crossover between the urinometers was made. Each patient had UO data registered hourly during daytime for 1, 2 or 3 consecutive days, with no minimum number of measurements required for inclusion in analysis.

The new AU calculates urine flow by measuring the change in height of a column of urine in a plastic chamber (Additional file 3). A capacitance-based sensor continuously registers the height of urine through the wall of the chamber. When filled, the chamber empties via a siphon. The computer unit of the urinometer is battery-powered and displays UO continuously. Data are displayed and stored on the unit and can optionally be transferred to a patient management system. The MU collects the urine in graded chambers, which allows for visual reading of the UO by the attending nurse. Measurements cannot be evaluated if the measuring setup is incorrectly positioned. The AU senses positions that interfere with measurements and displays an error message on its screen. The AU also displays an error message when the disposable unit needs to be replaced.

Directly after each hourly measurement with one of the urinometers, a laboratory technician made a standardized reference measurement of the urine volume with a laboratory precision measuring cylinder (250 ml, BLAUBRAND; BRAND GMBH + CO KG, Wertheim, Germany) with a tolerance of ±1 ml. The laboratory assistant was blinded to each urinometer measurement until after making the corresponding reference measurement, but was not blinded to the urinometer model. The urine volume remaining in the measuring chamber of the AU when reference measurements were made did generally change between two successive measurements. This affected the volume of the reference measurement, which therefore, only for the purpose of this study, necessitated adjustment by approximation of the urine volume in the measuring chamber at the time of each measurement. The approximation was made visually by the laboratory technician with the help of a transparent plastic measuring scale provided by the AU manufacturer (accuracy of ±1 ml).

Agreement evaluation of both urinometers compared with cylinder measurements was performed according to the methods devised by Bland and Altman [5,6] using *a priori* set limits of agreement, confidence intervals of obtained parameters and relationship analysis of differences and means. The bias and precision of the MU were used as *a priori* set acceptance limits for the AU. The independent samples *t*-test and Levene’s test were used to test for differences in bias and precision between the urinometers.

The absolute value of the deviation from exactly 1 hour between measurements was calculated and analyzed by mean, standard deviation and 95% confidence limits of agreement.

The temporal deviation of the MU was evaluated during a separate session at the ICU when the time of each measurement was noted.

The staff opinion of the urinometers was evaluated after completion of the urinometer measurement performance study. After a 15-minute theoretical introduction and a 3-day evaluation period of the AU at the ICU, all participating nurses filled out an anonymous questionnaire (Table 1) regarding how easy it was to learn to use the AU (question 1) and their opinion of the user-friendliness of the AU compared with the MU (questions 2 through 5). All answers were given on an ordinal scale from 1 to 5. Answers to question 1 were analyzed separately, whereas questions 2 to 5 were analyzed by aggregated mean and personal mean. A one-sample *t*-test was used to test for significance.

**Table 1:** **Staff opinion of an automatic urinometer compared with a manual urinometer**

**Question**	**Grading (n = 28 staff members)**				
	**5**	**4**	**3**	**2**	**1**
	*Very easy*	*Easy*	*Fair*	*Not easy*	*Hard*
1. How easy was it to learn to use the automatic urinometer?	39%	54%	7%	0%	0%
2. Was the collection of urine output data from the automatic urinometer easier compared with the manual urinometer?	32%	43%	14%	11%	0%
	*A lot less*	*Less*	*Same*	*More*	*Much more*
3. Did you feel that you had less contact with the urine bags with the automatic urinometer compared with the manual urinometer?	36%	25%	39%	0%	0%
	*Much more*	*More*	*Same*	*Less*	*Much less*
4. Do you think the reliability of the urine output data is higher with the automatic urinometer than with the manual urinometer?	21%	64%	7%	0%	7%
5. Does using the automatic urinometer give you more time for other activities?	0%	32%	68%	0%	0%

Statistical analysis was done in IBM SPSS Statistics software (IBM, Armonk, NY, USA). A *P*-value <0.05 was considered significant. The variables of the patient groups were compared by Student’s *t*-test when normally distributed and the Mann–Whitney *U* test when not so. Fisher’s exact test was used to compare binary group data.

## Results

**Figure 1:**
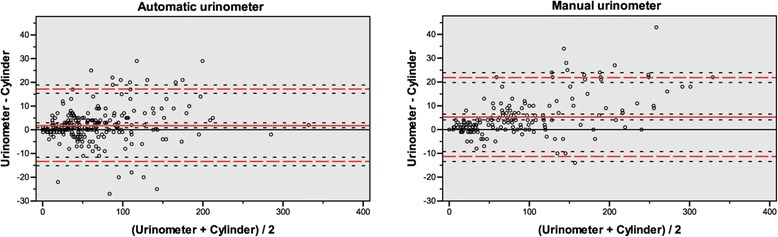
Bland-Altman plots of the agreement of each urinometer. Volumes are given in milliliters. In each plot, the red lines represent (from above): upper 95% limit of agreement; mean bias; lower 95% limit of agreement. Dotted lines represent confidence intervals of each parameter. Three measurements with *x*-axis volumes >400 ml were omitted for the purpose of visibility.

**Figure 2:**
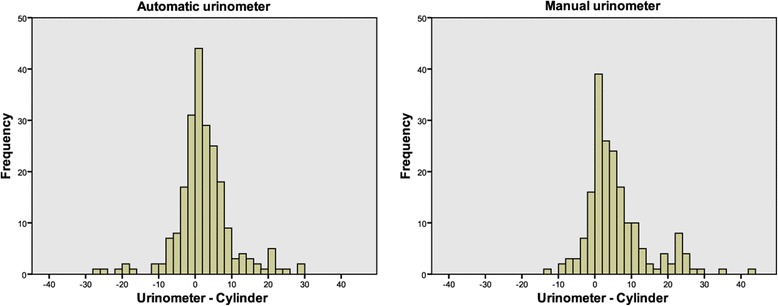
Histograms of the agreement of each urinometer. Volumes are given in milliliters. Each bar represents an increment of 2 ml. A positive value represents an overestimation by the urinometer compared with a reference measurement by measuring cylinder.

A total of 408 hourly UO measurements, 220 with the AU and 188 with the MU, from 34 included patients (Table 2) comprising 18 in the automatic group and 16 in the manual group were collected and analyzed. Of the collected measurements in the automatic group, 5.6% (13/233) were excluded from analysis, almost exclusively due to improper positioning of the unit after the patient had moved from the bed to a chair. The median (25th to 75th percentile range) number of UO measurements for each patient was similar for the two groups, with 10.5 (9.0 to 16.3) and 12.5 (8.3 to 14.8) for the AU and the MU, respectively. Each measurement was paired with a reference measurement conducted with a measuring cylinder. The mean of the cylinder measurements was 65 ml in the AU group and 96 ml in the MU group. Calculations (Table 3) and plots (Figure 1) made according to the method of Bland and Altman [5] showed a mean bias of +1.9 ml for the AU and +5.3 ml for the MU (*P* <0.0001). The standard deviations were 7.7 ml and 8.4 ml (*P* = 0.108), respectively, illustrated by 95% confidence intervals placed at ±15.2 ml and ±16.6 ml from the mean. The mean relative percentage deviations of the urinometers compared with their paired cylinder measurements were ±12.8% for the AU and ±12.7% for the MU (*P* = 0.94). The scatter of the AU had no evident change in bias with increasing urine volume (Figure 1) and showed approximate normality when plotted in a histogram (Figure 2). The scatter of the MU had a tendency of a larger positive bias with increasing urine volume (Figure 1) and showed a tendency toward skewness with a heavier and longer tail toward positive bias when plotted in a histogram (Figure 2).

**Table 2:** **Clinical variables of the patients in the automatic urinometer group and the manual urinometer group** ^**a**^

**Variable**	**AU (n = 18)**	**MU (n = 16)**	***P*** **-value**
Female sex, %	28	50	0.29
Age, yr	68.0 (64.3 to 75.5)	66.5 (63.5 to 73.5)	0.75
Weight, kg	79.2 ± 15.3	80.3 ± 7.9	0.83
Height, cm	174 ± 5.8	172 ± 9.8	0.48
BMI, kg/m^2^	1.9 ± 0.2	2.0 ± 0.2	0.74
EuroSCORE II, %	1.7 (0.9 to 2.6)	1.6 (0.9 to 3.1)	0.85
Preoperative albumin, g/L	39.0 (36.0 to 40.3)	38.0 (36.0 to 39.8)	0.60
Preoperative creatinine, μmol/L	82.5 (67.8 to 97.8)	86.5 (62.5 to 100)	0.83
Preoperative eGFR, ml/min	78.0 (62.4 to 104)	75.4 (67.8 to 89.4)	0.91
IDDM, %	6	6	1.00
COPD, %	6	6	1.00
LVEF <50%, %	33	31	1.00
Surgical procedures			
CABG, %	28	25	1.00
Single-valve replacement, %	44	44	1.00
Valve + CABG, %	11	19	0.65
Others, %	17	13	1.00
ECC, min	84.5 (71.5 to 133)	79.5 (67.0 to 127)	0.56
ICU stay, hr	22.5 (18.8 to 25.0)	23.0 (18.5 to 25.3)	0.77
Ventilation time in ICU, hr	3.0 (1.0 to 4.3)	2.5 (1.0 to 4.8)	0.96
Inotropes in ICU, %	0	6	0.47

^a^AU, Automatic urinometer; BMI, Body mass index; CABG, Coronary artery bypass graft; COPD, Chronic obstructive pulmonary disease; ECC, Extracorporeal circulation; eGFR, Estimated glomerular filtration rate (Cockcroft-Gault equation); EuroSCORE, European System for Cardiac Operative Risk Evaluation [8]; ICU, Intensive care unit; IDDM, Insulin-dependent diabetes mellitus; LVEF, Left ventricular ejection fraction; MU, Manual urinometer. Data are presented as percentages, medians (25th to 75th percentile range) or means ± standard deviation.

**Table 3:** **Performance parameters of the automatic urinometer and the manual urinometer** ^**a**^

					**Upper LOA**			**Bias**			**Lower LOA**		
**Urinometer parameters**		**n**	**SD**	**SE**	**CI+**	$\overline{\mathbf{x}}$	**CI−**	**CI+**	$\overline{\mathbf{x}}$	**CI−**	**CI+**	$\overline{\mathbf{x}}$	**CI−**
AU	All	220	7.7	0.5	+18.9	+17.1	+15.4	+2.9	+1.9	+0.9	−11.6	−13.3	−15.1
	<100 ml	176	6.4	0.5	+15.4	+13.8	+12.2	+2.2	+1.3	+0.3	−9.7	−11.3	−12.9
	≥100 ml	44	11.4	1.7	+33.5	+27.6	+21.6	+8.0	+4.5	+1.0	−12.6	−18.6	−24.5
MU	All	188	8.4	0.6	+23.9	+21.8	+19.8	+6.5	+5.3	+4.1	−9.2	−11.3	−13.4
	<100 ml	124	4.5	0.4	+13.1	+11.6	+10.4	+3.6	+2.8	+2.0	−4.8	−6.2	−7.6
	≥100 ml	64	11.5	1.4	+38.1	+33.1	+28.2	+13.0	+10.1	+7.2	−8.0	−12.9	−17.9

^a^AU, Automatic urinometer; CI, Confidence interval; LOA, Limit of agreement; MU, Manual urinometer; n, Number of measurements; SD, Standard deviation; SE, Standard error; $\overline{\mathrm{x}}$, Mean. For each urinometer, data are shown for all measurements combined, as well as subdivided at a volume of 100 ml. All parameters are in milliliters, except n.

Among all 408 measurements, 146 (36%) were identified with a UO of <40 ml/hr by either the used urinometer or the reference measurement (Table 4). The AU had a sensitivity of 90%, a specificity of 99%, a positive predictive value of 97% and a negative predictive value of 94%. The MU had a sensitivity of 98%, a specificity of 96%, a positive predictive value of 92% and a negative predictive value of 99%. The number of discrepancies between the urinometers and the gold standard were few, which gave a large variability of the parameters.

**Table 4:** **Evaluation of diagnostics of urine output <40 ml/hr** ^**a**^

**Urinometer**	**AU**	**MU**
Sensitivity (%)	90.4	98.2
Specificity (%)	98.5	96.2
Positive predictive value (%)	97.4	91.7
Negative predictive value (%)	94.4	99.2

^a^The automatic urinometer (AU) and manual urinometer (MU) are compared with a measuring cylinder (gold standard).

The times between two consecutive measurements were recorded, and the absolute difference from precisely 1 hour was calculated (n = 108). The mean temporal variation with the MU was ±7.4 minutes (±12.4%), and the 95% limit of agreement was ±23.9 minutes (±39.8%), compared with no temporal variation with the AU (*P* <0.0001).

The 28 participating nurses completed the questionnaires (Table 1). Ninety-three percent of the nurses found the AU to be either easy or very easy to learn to use (question 1). The aggregate mean score of questions 2 to 5 was 3.8 (standard deviation (SD) ±0.9) (*P* <0.0001 compared with mean = 3), with 86% of the nurses considering the AU superior to the MU (personal mean >3) (*P* <0.0001). Of the answers, 63% were in favor of the AU, 5% were in favor of the MU and 32% graded them as equal.

## Discussion

When evaluating a new measuring system, one has to compare it with other standard methods, preferably also with the gold standard. Thus, every patient was allotted to an hourly measurement with the gold standard as well as either the AU or the MU. This study design enabled us to compare the performance AU with that of the MU, which is used in the wards of our hospital today. In short, the new automatic UO system, based on capacitance measurements, was not inferior to its manual counterpart and had significantly better results in terms of bias, temporal deviation and staff opinion.

This study showed a significantly lower bias of the AU compared with the MU. When extrapolating this over the course of 24 hours, the daily biases would be 46 ml and 126 ml, respectively, which may be considered acceptable in clinical practice, although smaller biases are preferable. The level of precision, which did not differ significantly between the urinometers, was also found to be acceptable because single errors of this magnitude would not, in our view, alter clinical decision making. The AU and the MU were, in our opinion, equally efficient in identifying cases of oliguria, although the study group sizes were too small to give definitive answers in this regard.

Another source of error when evaluating UO monitoring, and which to our knowledge has not been assessed before, is the temporal deviation of measurements with a MU, which may further lower its precision. This error could in theory be avoided only if the patient data management system were programmed to make corrective calculations. In contrast, this source of error is inherently avoided with an automatic continuous measurement device.

Introduction of new equipment in a ward necessitates the education and acceptance of the ward staff. The staff opinion of the new equipment is therefore of utmost importance. The study started with a standard, brief, 15-minute theoretical introduction to the equipment, followed by a 3-day evaluation period. Thereafter, the staff evaluated the devices and found the AU to be easy to learn to use and gave the AU a significantly higher rating than the MU. Although significantly higher, the rating of the AU compared with the MU was not remarkably high. This result may be expected, as the model of the AU used in this study did not automatically enter the UO data into the patient data management system. Automatic data entry, when activated, may possibly raise the score of questions 2, 3 and 5 (Table 1), which would suggest a further increase in staff satisfaction with the AU.

A disadvantage with the MU occurs during high UO volumes. If the UO during 1 hour exceeds 500 ml, the MU will not be able to incorporate the whole volume in the measurement chambers, but will divert the excess into the urine bag. Therefore, these higher values can be estimated only if the urine bag is emptied hourly. However, in our study, this problem did not occur, because, according to the protocol, the urinary bag was emptied hourly.

In Figure 1, the greater spread of the scatter at higher volumes in both plots signals a lower precision at higher volumes. In the AU, this may result from a small miscalculation repeated in each siphon cycle, whereas in the MU this is expected because of the less fine grading of the MU at higher volumes. If a Bland-Altman plot diverges at higher means, the ratio of difference and mean can be plotted on the *y*-axis instead of the difference [5]. We considered such an approach inappropriate for this study because such a correction would have shifted the error from large absolute deviations among high means to large relative deviations among small means. The use of absolute values in the regular clinical setting also made the use of absolute values more appropriate. The tendency toward skewness in the histogram of the MU (Figure 2) may indicate that the visual reading of its analogue scale generally overestimates the UO.

An earlier study by Hersch *et al*. [7], who used a similar study design, evaluated another automatic UO measurements system, based on droplet counting. They found the droplet-based AU superior to its MU counterpart in terms of bias, precision and user-friendliness. With regard to comparison of the conductance and droplet-based automatic systems, the following comments can be made. First, the biases and precisions were +1.9 ml (SD ±7.7 ml) and +0.08 ml (SD ±14 ml), respectively. These values are, in our opinion, comparable. Second, the biases of the MU in our studies were +5.3 ml (SD ±8.4 ml) and +13 ml (SD ±68 ml), respectively. The much higher values for the MU reported by Hersch *et al*. [7] are difficult to explain. Although the brand of the MU was the same in both studies, it is unclear if the same models were used, as Hersch *et al*. [7] did not clearly state which model they used in their study.

The clinical implications of an AU can be discussed. Inherent with the use of an MU is that the staff needs to read and empty the system manually at exact time intervals. This is usually unfeasible, but if the staff density indeed allows this, our study indicates the MU to be acceptable for hourly UO measurements and comparable to the AU in measurement performance. However, regardless of the staff density, use of the AU may open up time for the staff to attend to other tasks. Furthermore, the AU may allow for hourly UO measurements on normal wards, where it is usually measured only a few times daily. Another advantage with the AU may be a possible decrease in contamination, given that direct contact with the system can be minimized and that the staff does not need to crouch down to do the reading.

There are some limitations of our study that may have affected our results. First, our study was carried out during two time periods; the MU was evaluated first and the AU thereafter. Thus, our patients were not randomized, but rather were allotted to the urinometer currently in use. A second limitation was a difference in the mean of the hourly urine cylinder reference measurements between the two urinometer groups, with the higher mean value in the manual group. This may have affected our results, but subgroup analyses done by delimiting each urinometer group at hourly UO values of 100 ml (Table 3) gave similar results in both ranges compared with the overall result. Also, we excluded measurements if the measuring setup had accidentally been incorrectly positioned, which occurred almost exclusively when the patient had been moved from the bed to a chair. This should be avoidable by further education of the ward staff on how to correctly position the AU. Similar errors may occur with the MU, although these are probably not perceived as clearly as with the AU.

## Conclusions

In this study, we compared the performance of a new capacitance-based AU with that of a standard MU in an ICU setting. The AU was not inferior to the MU and significantly better than the MU in terms of bias, temporal deviation and staff opinion, although the clinical relevance of these findings may be open to discussion.

## Key messages

- A new capacitance-based automatic urinometer was compared with a standard manual urinometer in an ICU setting and was found not to be inferior.
- The automatic urinometer was significantly better than its manual counterpart in terms of bias, temporal deviation and staff opinion, although the clinical relevance of these findings may be open to discussion.
- The automatic urinometer may open up time for staff to attend to other tasks.
- The automatic urinometer may allow for hourly urinary output measurements on normal wards, where UO is usually measured only a few times daily.
- Use of the automatic urinometer may possibly decrease contamination, given that direct contact with the system can be reduced and that the staff do not need to crouch down to take readings.

## Additional files

Additional file 1:
**Overview of the automatic urinometer.**
Additional file 2:
**Screen of the automatic urinometer.**
Additional file 3:
**Measuring chamber of the automatic urinometer.**

## Abbreviations

AU: Automatic urinometer
BMI: Body mass index
CABG: Coronary artery bypass graft
CI: Confidence interval
COPD: Chronic obstructive pulmonary disease
ECC: Extracorporeal circulation
eGFR: Estimated glomerular filtration rate (Cockcroft-Gault equation)
EuroSCORE: European System for Cardiac Operative Risk Evaluation
ICU: Intensive care unit
IDDM: Insulin-dependent diabetes mellitus
LOA: Limit of agreement
LVEF: Left ventricular ejection fraction
MU: Manual urinometer
SD: Standard deviation
SE: Standard error
UO: Urine output
