# Supplementary figures and images for: Performance and user evaluation of a novel capacitance-based automatic urinometer compared with a manual standard urinometer after elective cardiac surgery
Source: Crit Care. 2015 Apr 21;19(1):173. doi: 10.1186/s13054-015-0899-4 (PMC4416393; doi:10.1186/s13054-015-0899-4)

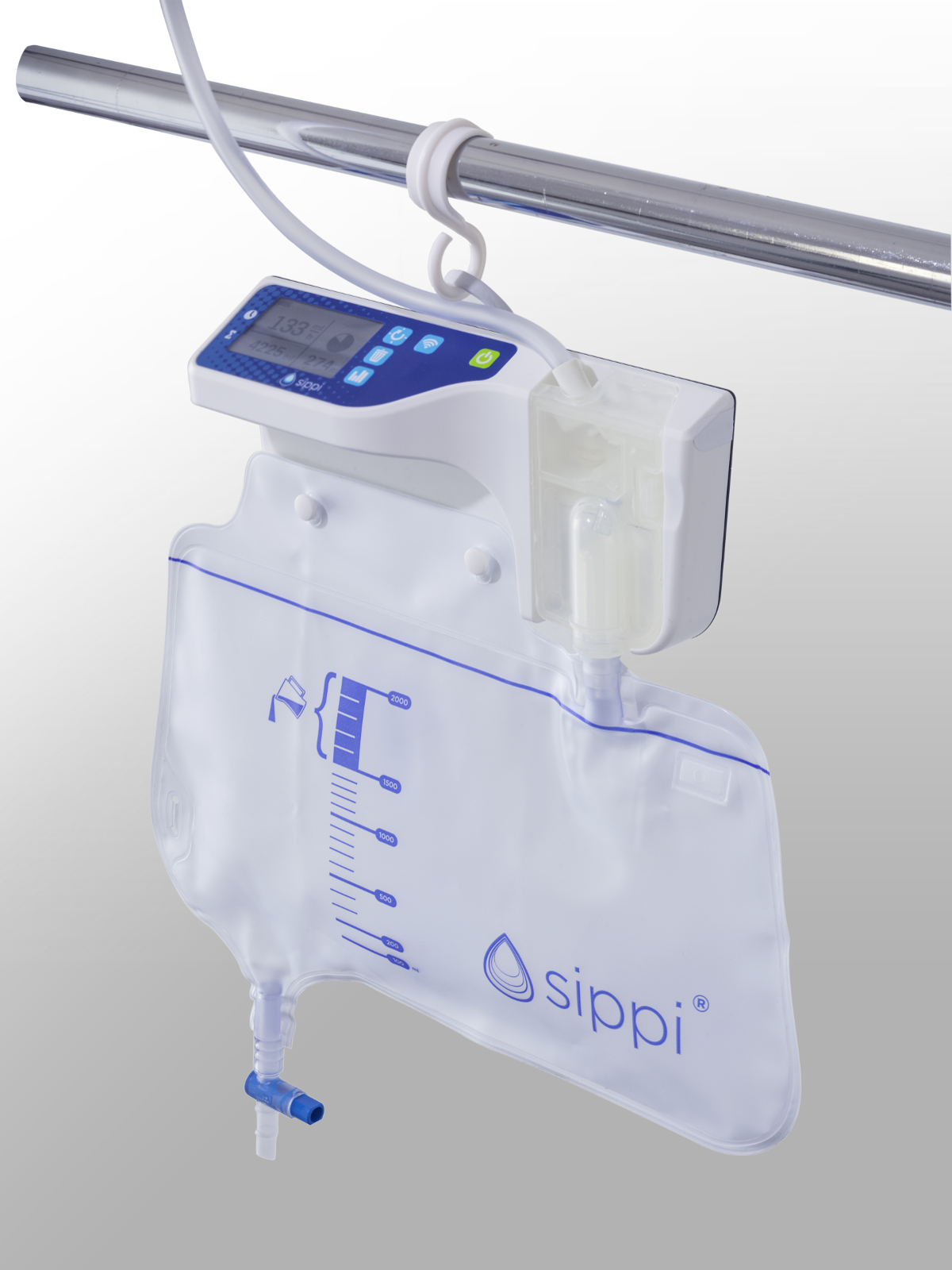

Supplement: Additional file 1: — Overview of the automatic urinometer. [file 13054_2015_899_MOESM1_ESM.jpeg]

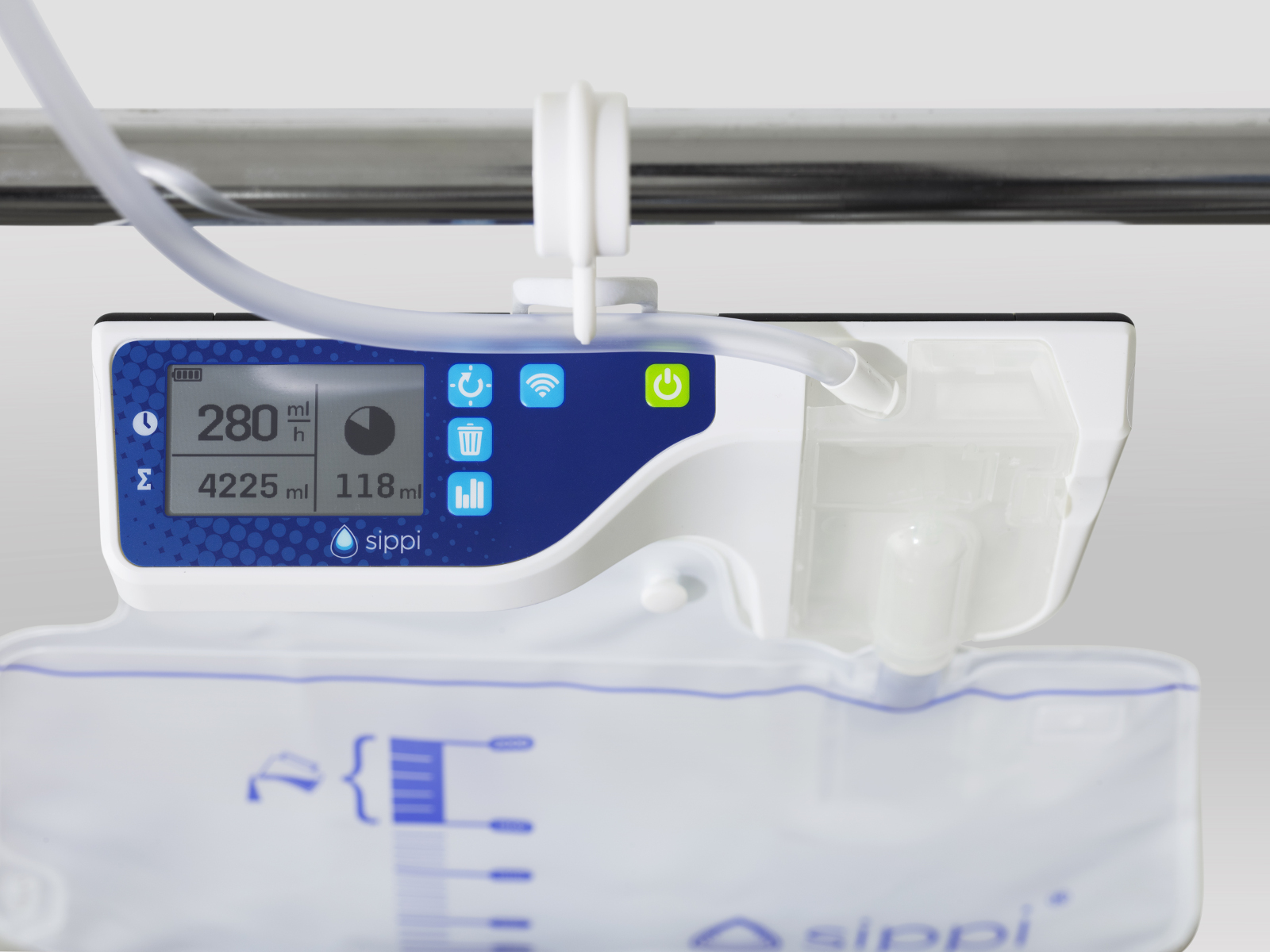

Supplement: Additional file 2: — Screen of the automatic urinometer. [file 13054_2015_899_MOESM2_ESM.jpeg]

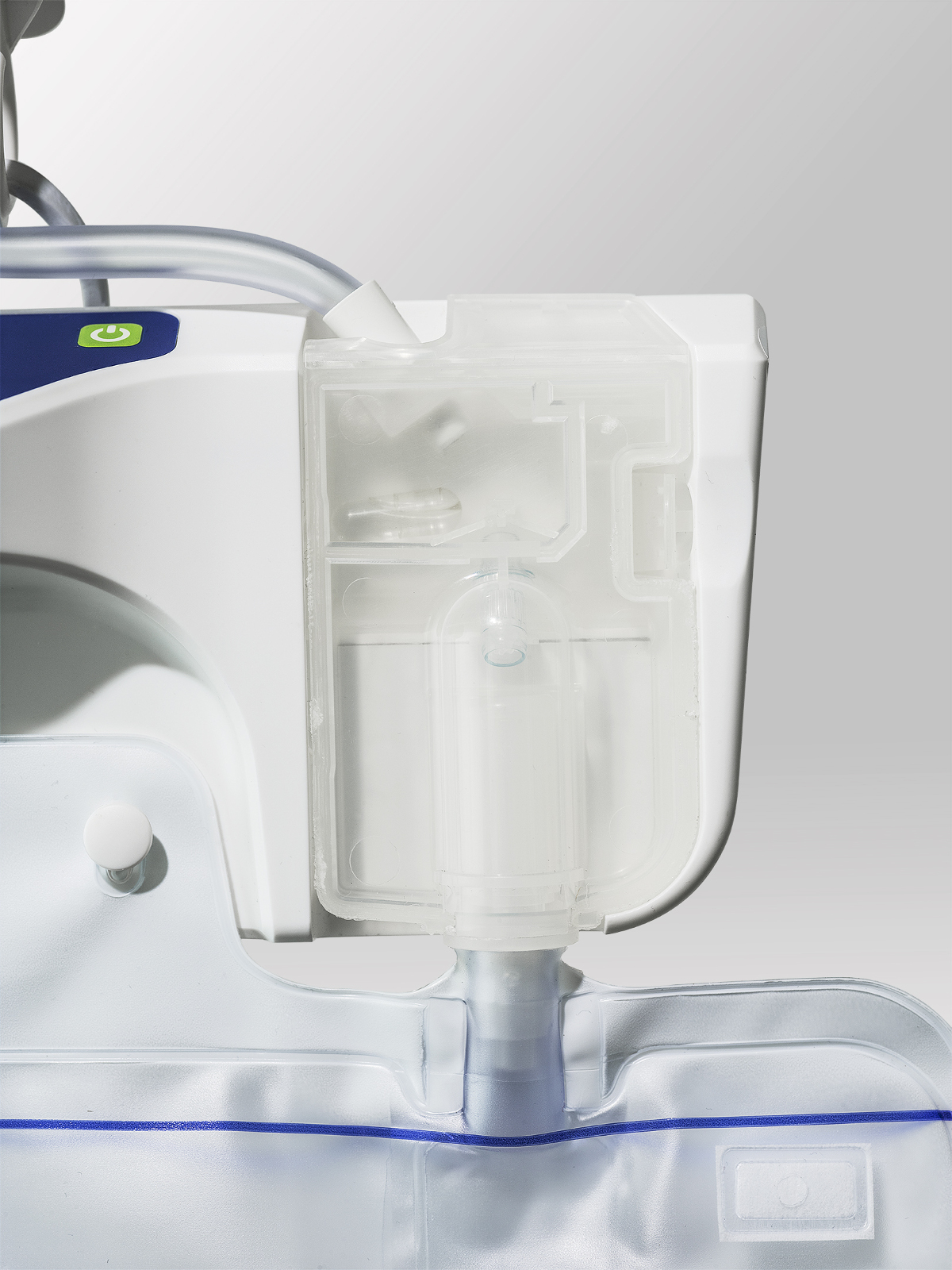

Supplement: Additional file 3: — Measuring chamber of the automatic urinometer. [file 13054_2015_899_MOESM3_ESM.jpeg]
